# Silicon-mediated alleviation of cadmium stress in *Pennisetum glaucum*: insights from physiology and root transcriptomics analysis

**DOI:** 10.3389/fpls.2026.1916768

**Published:** 2026-10-13

**Authors:** Taowen Pan, Qiyu Dong, Bo Li, Abdul Hafeez, Yixia Cai, Kunzheng Cai, Jibiao Geng

**Affiliations:** 1 College of Resources and Environment, Linyi University, Linyi, Shandong, China; 2 Key Laboratory of Green Functional Fertilizer Innovation, Ministry of Agriculture and Rural Affairs, Linyi University, Linyi, Shandong, China; 3 College of Natural Resources and Environment, South China Agricultural University, Guangzhou, Guangdong, China; 4 Key Laboratory of Tropical Agro-Environment, Ministry of Agriculture and Rural Affairs, South China Agricultural University, Guangzhou, Shandong, China; 5 College of Resources and Environment, Baoshan University, Baoshan, Yunnan, China

**Keywords:** cadmium, pearl millet, phytoremediation, silicon, transcriptomics

## Abstract

The problem of cadmium (Cd) pollution in soil has attracted much attention. Although *Pennisetum glaucum* has developed tolerance mechanisms to heavy metals, the accumulation of excess Cd through the food chain threatens human health. Silicon (Si) has a beneficial role in alleviating environmental stress. However, the underlying physiological and molecular mechanisms for alleviating Cd toxicity in *P. glaucum* are still largely unknown. Therefore, experiments were conducted to explore the effects of Si on the photosynthetic system, antioxidant defense system, and the expression of in *P. glaucum* under Cd stress. The results showed that Si amendment increased the net photosynthetic rate and chlorophyll content of Cd-stressed *P. glaucum* by 19.7% and 13.8%, respectively. Moreover, DHAR and MDHAR activities in roots were significantly increased by 53.6% and 63.6% in the AsA-GSH cycle after Si application, while MDA concentration was significantly decreased by 14.6%. Importantly, exogenous Si application dramatically reduced Cd concentration in roots and shoots of *P. glaucum* by 52.7% and 21.5%, respectively under Cd stress. RNA-seq analysis showed that 6491 differentially expressed genes (DEGs) were identified, among which 4429 DEGs were down-regulated and 2062 DEGs were up-regulated. Root transcriptomic analysis revealed that gene expression differed between the Cd and Cd+Si treatments, particularly for genes related to photosynthesis and glutathione metabolism. Contrarily, the expression of genes involved in Cd uptake and transport, such as *ABCG36*, *ABCB11*, *Nramp5*, and *Nramp4* (folds were -1.10, -2.52, -2.66, and -1.55, respectively) were down-regulated by the addition of Si. Overall, these above results demonstrate that Si supplementation offers a viable approach to reduce Cd accumulation and ensure the safe production of forage crops.

## 1 Introduction

Heavy metal (HM) pollution has become an urgent environmental problem worldwide to be solved (Wang et al., 2022; Guo et al., 2024). Cadmium (Cd) has gained immense attention as a result of its potent toxicity and non-reversibility (Haider et al., 2021; Zhao et al., 2025). Plants can adopt different strategies to defend against Cd toxicity, such as activation of antioxidant enzyme systems, the response of transporters, osmoregulation, and gene regulation (Al-Khayri et al., 2023). However, excessive Cd affects the normal physiological processes of plants, eventually leading to the decrease of photosynthetic rate, accumulation of reactive oxygen species, and disorder of gene expression, etc (Huybrechts et al., 2019; Liu et al., 2023a). In addition, Cd inhibits enzyme activity in cells by combining with sulfhydryl (-SH) in proteins (Qi et al., 2025), and disrupts ion absorption processes by competing with essential metals such as calcium and magnesium for entry channels (Cai et al., 2022). More seriously, excessive Cd can cause various human diseases through the food chain. Therefore, it is necessary to find an environmentally friendly and effective method to reduce the accumulation of Cd in plants.

Nowadays, many strategies have been applied to remediate Cd-contaminated soils and reduce Cd accumulation in plants, such as the application of soil amendments, water management, and the cultivation of low-Cd-accumulating crop varieties (Haider et al., 2021; Mei et al., 2022). Among them, the addition of beneficial and essential elements to contaminated soils is considered an environmentally friendly strategy for remediating Cd-polluted soils (Jiang et al., 2023). As the second most abundant element in the earth’s crust, silicon (Si) in soil is mainly absorbed and utilized by plants in the form of monosilicic acid (Pan et al., 2023). Studies have shown that the role of Si in enhancing plant stress resistance has been well recognized (Yang et al., 2021; Alam et al., 2025). Importantly, Si fertilization is an important way to improve crop yield and safety in contaminated soils (Zhao et al., 2022). Furthermore, the possible mechanisms by which Si reduces Cd uptake and translocation in plants have been studied in various plant species. For instance, Cai et al. (2022) reported that split application of Si at both transplanting and jointing stages significantly enhanced Cd sequestration in rice roots by promoting Cd distribution to cell walls, increasing the chelation of Cd with thiol compounds and polysaccharides, and down-regulating the expression of *OsNramp1* and *OsNramp5* genes. In addition, alleviative effects of Si on Cd stress have also been observed in wheat and rapeseed, primarily involving the reduction of phytoavailable Cd in soil and the regulation of cellular oxidative stress metabolism (Alia et al., 2019; Ahmed et al., 2023). Overall, the mechanisms by which Si alleviates Cd toxicity mainly include: activation of antioxidant defense systems, chelation and precipitation of heavy metals, Cd compartmentalization in vacuoles, and regulation of Cd-related gene expression (Liu et al., 2023b; Riaz et al., 2023; Islam et al., 2024).

*Pennisetum glaucum* is often used as silage and green manure because of its rich nutrition, fast growth, large biomass, and strong resistance (Khan et al., 2023). Moreover, *P. glaucum* has high tolerance to heavy metals and is commonly used to remediate Cd pollution in the environment (Essid et al., 2026). However, Cd concentrations in grass sprouts exceed the maximum tolerated levels of Cd in animal diets, which may ultimately cause irreversible damage to human kidney function through the food chain (Awan et al., 2023). Moreover, there are few studies on the safety evaluation of *P. glaucum* in heavy metal-contaminated soils. Therefore, analysis of heavy metal Cd in forage grasses is important to improve environmental pollution. Nowadays, Transcriptome analysis is a combination of gene information and biological functions, which contributes to the discovery of gene regulatory mechanisms related to stress adaptation (Yang et al., 2024). Transcriptome analysis of rice seedling roots by Zhao et al. (2023) showed that Cd-induced differentially expressed genes (DEGs) were mainly related to biological processes such as signal transduction, ion transport, and ROS scavenging. Chen et al. (2021) explained the key genes and pathways of kenaf with different Cd tolerance by comparing transcriptomes. However, most studies on Cd tolerance have focused on food crops such as rice, wheat, and maize, leaving the forage crop *P. glaucum* largely unexamined, particularly at the molecular level. This research gap has not only impeded our understanding of the mechanisms by which Si enhances Cd tolerance in *P. glaucum* but has also hampered the development of effective safe utilization strategies for Cd-contaminated soils.

The purpose of this study was to explore the mechanisms behind the beneficial effects of exogenous Si on *P. glaucum* under Cd stress. Therefore, we will focus on the following two aspects in this study: (1) the effects of Si application on the photosynthetic system and antioxidant enzyme activities (AsA-GSH cycle) under Cd stress; (2) the expression of Cd-responsive genes and pathways mediated by Si addition under Cd stress. The results would expect to provide important information basis for the identification of Cd stress-related genes and further elucidate the underlying physiological and molecular mechanisms of Si-mediated Cd resistance in *P. glaucum*.

## 2 Materials and methods

### 2.1 Experimental materials and design

Pot experiment was conducted in Ecological Farm of South China Agricultural University (113°21′ E, 23°09′ N), and *P. glaucum* was selected as the experimental material. The sterilized *P. glaucum* seeds were placed in an incubator at 28 °C (under dark conditions) to accelerate germination for 24 hours, and then evenly sprinkled into plastic pots containing 2.5 kg of soil. During the experiment, urea and potassium dihydrogen phosphate were supplemented according to the growth of *P. glaucum*. The basic characteristics of the tested soils were listed in **Supplementary Table 1**.

Four treatments with three biological replicates were designed in the experiment, including: (1) no addition of Cd and Si (CK); (2) the addition of 2 mmol kg ^-1^ Si in the form of K_2_SiO_3_ (Si); (3) the addition of 50 mg kg^-1^ Cd in the form of CdCl_2_ (Cd), and (4) the addition of both 50 mg kg^-1^ Cd and 2 mmol kg^-1^ Si (Cd+Si). The effect of potassium was eliminated by adding the same amount of potassium chloride solute ion. There were six *P. glaucum* seedlings for each pot. Plant samples were collected at 60 days after the addition of CdCl_2_ and K_2_SiO_3._ Photosynthetic and physiological indicators of *P. glaucum* were measured, and the roots were sampled and frozen separately in a -80 °C refrigerator for the determination of malondialdehyde (MDA), antioxidant enzyme activities and transcriptome analysis, respectively. In addition, dried plant samples were used to determine the concentrations of Si and Cd in roots and shoots of *P. glaucum*.

### 2.2 Determination of photosynthesis parameters

Determination of photosynthesis-related indicators on the first fully expanded leaf with a photosynthetic instrument (Li-6400XT, USA). The light intensity and flow rate to the sample cell were set as 1000 μmol m^-2^ s^-1^ and 500 μmol s^-1^, respectively. Each sample was replicated three times.

### 2.3 Determination of chlorophyll fluorescence parameters

Chlorophyll fluorescence parameters in leaves of *P. glaucum* were measured (dark treatment for 20 min) using the light induction curve program of the chlorophyll fluorometer (MINI-PAM-II, Germany). The parameters include photochemical quenching (*qP*), non-photochemical quenching (*NPQ*), maximum yield of the photosystem II (*F*v/*F*_m_), and potential yield of the photosystem II (*F*v/*F*_0_). In addition, chlorophyll content in leaves was measured by a chlorophyll meter (SPAD-502, Japan).

### 2.4 Determination of oxidative stress and antioxidant enzyme activity

Fresh root tissue samples (0.5 g) were ground and homogenized with 5 mL phosphate buffer (pH 7.8) in an ice bath. The extract was centrifuged at 5000 r min^-1^ for 20 min at 4 °C, and the supernatant was used for the determination of malondialdehyde (MDA), ascorbate peroxidase (APX), dehydroascorbate reductase (DHAR), and monodehydroascorbate reductase (MDHAR).

The determination of MDA content was based on the thiobarbituric acid colorimetric method (Wu et al., 2017). The activity of APX was measured based on the method by Nakano and Asada (1987), and the amount of oxidized 1 moL min^-1^ ascorbic acid (AsA) was defined as a unit of enzyme activity (U). In addition, the activities of DHAR and MDHAR were measured at 265 nm and 340 nm according to the methods described by Hossain et al. (1984) and Nakano and Asada (1981), respectively.

### 2.5 Determination of silicon and cadmium concentration

The concentration of Si in roots of *P. glaucum* was determined by high temperature alkali fusion (Elliott and Snyder, 1991). In brief, 0.1 g of dry root tissue samples were mixed with 3 mL of 50% sodium hydroxide and then autoclaved. The reaction system included: 1mL supernatant (diluted 50 times), 30 mL 20% glacial acetic acid, 10 mL ammonium molybdate solution, 5 mL 20% tartaric acid, and 1mL reducing agent. The absorbance value was measured at 650 nm to calculate the Si concentration of the sample. Finally, the absorbance value was measured at 650 nm to calculate the Si concentration of the sample.

According to the method of Zhang et al. (2014), 0.2 g of dry root tissue samples were digested in a microwave digestion instrument (nitric acid and hydrogen peroxide), and the Cd concentration in roots of *P. glaucum* was measured in a flame atomic absorption instrument.

### 2.6 Root transcriptome analysis

Numerous studies have shown that Si does not influence plant growth and defense response under no-Cd stress, so we focus on the transcriptome analysis of *P. glaucum* roots mediated by Si under Cd stress. The total RNA of *P. glaucum* root was extracted by TRIzol reagent method, and the RNA quality was detected by NanoDrop 2000 (Thermo Fisher Scientific, USA). Briefly, high-throughput sequencing was performed after the enriched mRNA fragments were used as templates to synthesize cDNA strands. After the library was constructed, the effective concentration of the library was accurately quantified using a real-time quantitative method (the effective concentration of the library is > 2 nM) to ensure the quality of the library. The constructed library was paired-end sequenced using Illumina HiSeq™ 4000 with a paired-end read length of 100 bp.

Unigene expression level was calculated by reads per kilobase per million mapped reads (RPKM), and differentially expressed genes (DEGs) were selected by |log2-fold change| ≥ 1 and false discovery rate (FDR) < 0.05. Finally, functional annotation and metabolic pathway analysis of DEGs were performed by Gene Ontology (GO) and Kyoto Encyclopedia of Genes and Genomes (KEGG).

### 2.7 Quantitative real-time PCR analysis

In order to ensure the reliability of the transcriptome data, eight candidate genes of different pathways were verified. The extraction method of RNA was the same as that of the transcriptome. Afterwards, the RNA was transcribed using a reverse transcription kit R223 (Vazyme, CHN) and amplified by PCR. The amplification program and the primers used for detection were shown in **Supplementary Table 2**.

### 2.8 Statistical analysis

Physiological and root transcriptome data in the experiment were analyzed by one-way analysis of variance (ANOVA) and independent sample t-test, respectively. In addition, a p-value less than 0.05 indicated a significant difference. Origin 2025 and Adobe Illustrator 2019 were used for drawing.

## 3 Results

### 3.1 Photosynthesis parameters

Cd stress severely inhibited the photosynthesis of *P. glaucum*, which was reflected in the reduction of net photosynthetic rate (*P*_n_), transpiration rate (*T*r), stomatal conductance (*G*s), and intercellular CO_2_ concentration (*C*_i_) (**Figure 1**). However, these adverse effects can be mitigated by Si modification. Without Cd stress, Si application significantly increased *P*_n_ and *C*_i_ of *P. glaucum* leaves by 8.9% and 33.3%, but had no effect on other photosynthesis-related parameters. In addition, Si application significantly increased *P*_n_, *T*_r_, *G*_S_, and *C*_i_ of Cd-stressed *P. glaucum* leaves by 19.7%, 54.1%, 49.4%, and 43.8%.

**Figure 1:**
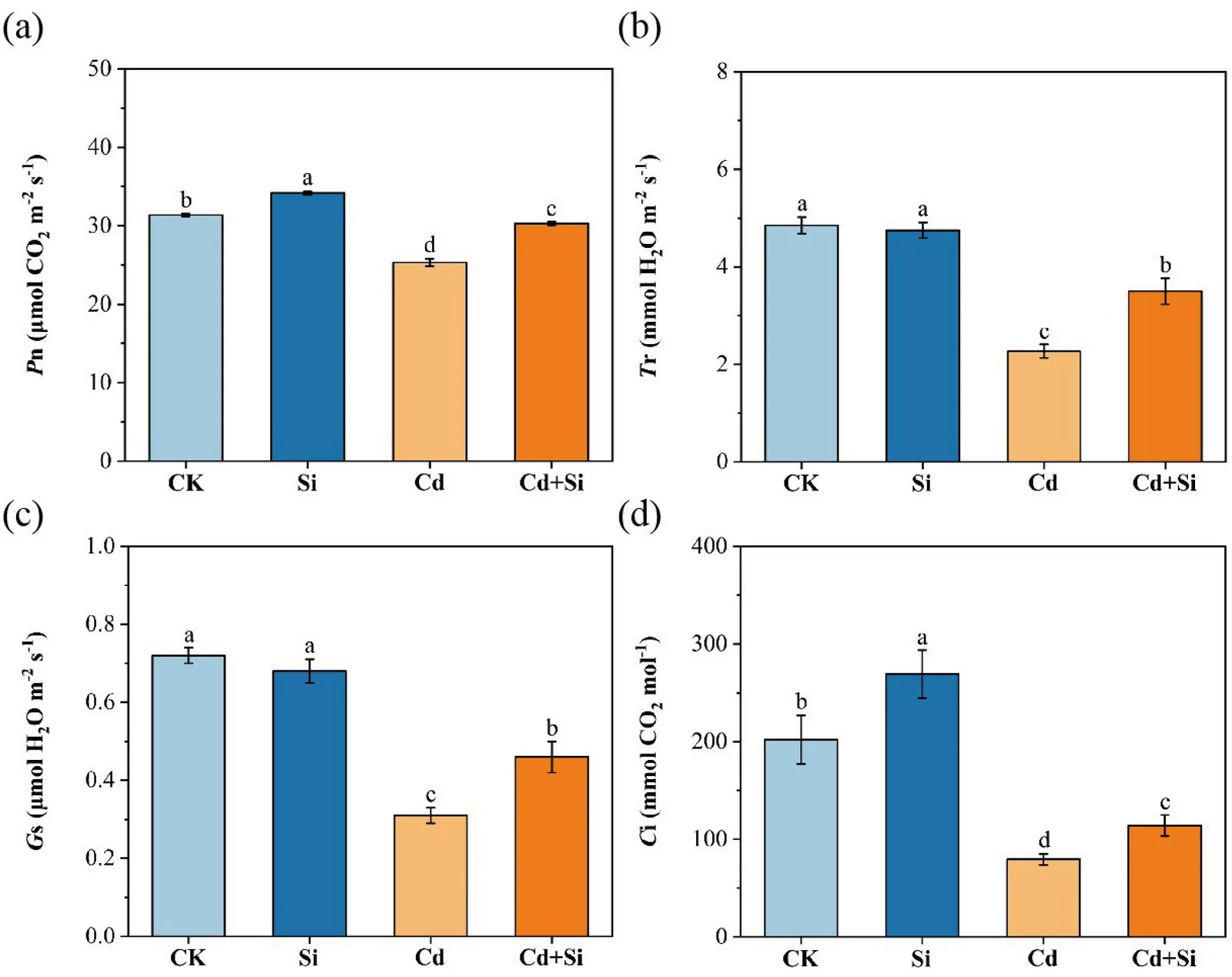
Effects of Si application on photosynthetic rate **(a)**, transpiration rate **(b)**, stomatal conductance **(c)** and intercellular CO_2_ concentration **(d)** of *P. glaucum* under Cd stress. All values are presented as the mean ± standard deviation (n = 3), and significant differences among treatments are indicated by letters (*p* < 0.05).

### 3.2 Chlorophyll fluorescence parameters

Compared with CK, Cd treatment significantly decreased the value of *F*v/*F*_m_ and *F*v/*F*_0_ in leaves (**Table 1**). Without Cd stress, Si application had no effect on the chlorophyll fluorescence parameters. However, Si addition significantly increased the value of *F*v/*F*_m_ and *F*v/*F*_0_ by 3.75% and 17.65%, respectively under Cd stress. Moreover, the chlorophyll content was also significantly raised by 13.8% after Si application.

**Table 1:** Effects of Si application on chlorophyll fluorescence parameters in leaves under Cd stress.

Treatment	*qP*	*NPQ*	*F*v/*F*_m_	*F*v/*F*_0_	Chlorophyll
CK	0.59 ± 0.05 a	1.41 ± 0.05 a	0.83 ± 0.00 a	4.88 ± 0.15 a	41.30 ± 0.64 a
Si	0.59 ± 0.06 a	1.65 ± 0.26 a	0.83 ± 0.01 a	4.88 ± 0.19 a	41.46 ± 0.84 a
Cd	0.61 ± 0.02 a	1.52 ± 0.11 a	0.80 ± 0.01 b	4.08 ± 0.20 b	33.73 ± 0.79 c
Cd+Si	0.55 ± 0.05 a	1.26 ± 0.09 a	0.83 ± 0.01 a	4.80 ± 0.22 a	38.38 ± 0.29 b

All values are presented as the mean ± standard deviation (n = 3), and significant differences among treatments are indicated by letters (*p* < 0.05).

### 3.3 Malondialdehyde concentration and antioxidant enzyme activity

The MDA concentration in plant cells reflects the degree of oxidative response to stress. Among all treatments, the highest MDA concentration in roots of *P. glaucum* under Cd treatment was 5.95 μmol g^-1^ (**Figure 2a**). Under Cd stress, Si effectively reduced the concentration of MDA in Cd-stressed roots of *P. glaucum* by 14.6%.

**Figure 2:**
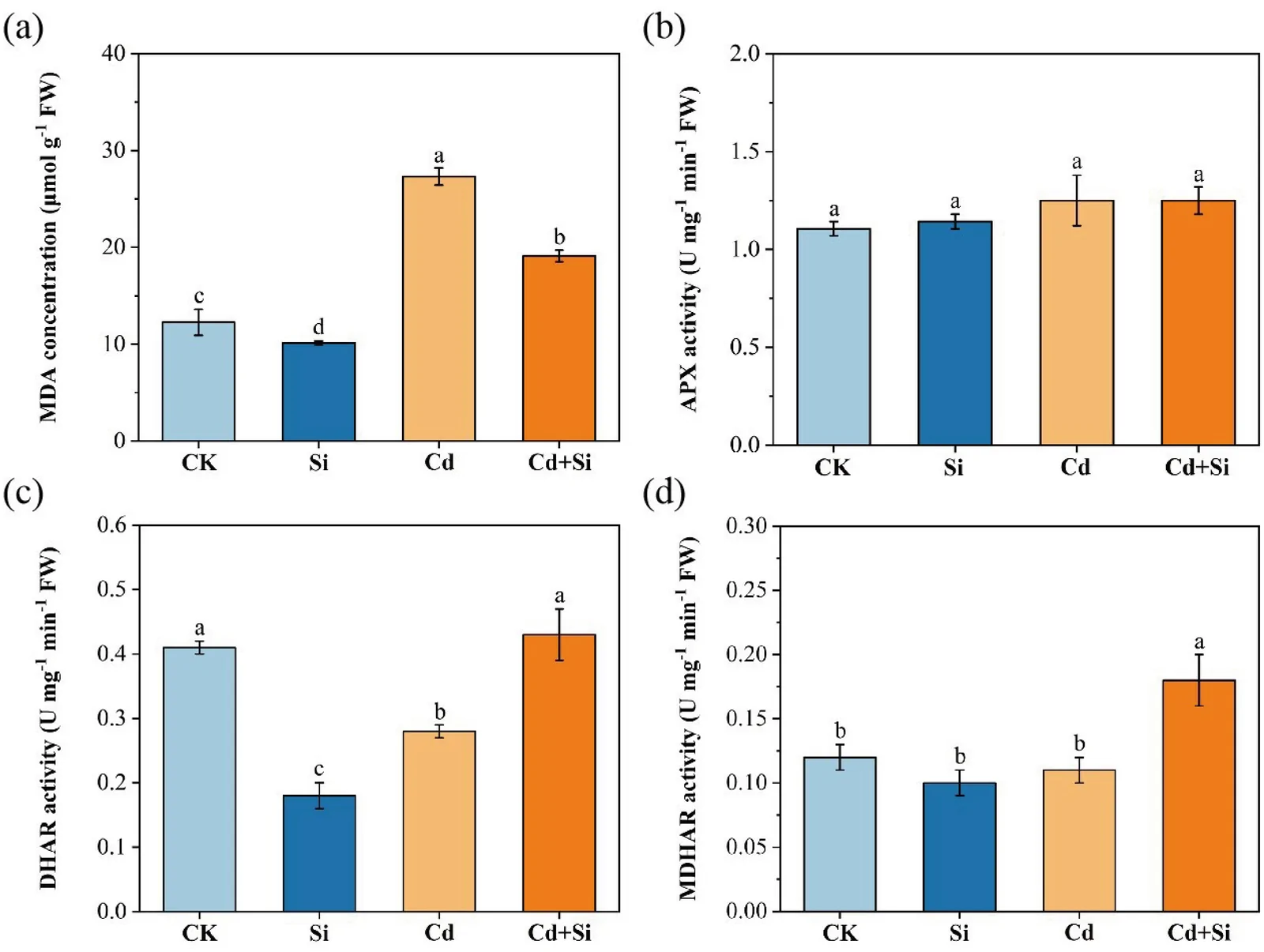
Effects of Si application on malondialdehyde **(a)**, ascorbate peroxidase **(b)**, dehydroascorbate reductase **(c)**, and monodehydroascorbate reductase **(d)** in roots of *P. glaucum* under Cd stress. All values are presented as the mean ± standard deviation (n = 3), and significant differences among treatments are indicated by letters (*p* < 0.05).

Without Cd stress, Si application had no influence on the activities of APX and MDHAR in *P. glaucum* roots (**Figures 2b, d**). Compared with CK, Cd stress significantly reduced the activity of DHAR by 32.4% (**Figure 2c**). However, Si addition increased the activities of DHAR and MDHAR in Cd-stress roots by 63.6% and 53.6%, respectively.

### 3.4 Concentrations of silicon and cadmium

Under Cd stress, Si application significantly increased the concentration of Si in roots and shoots of *P. glaucum* (**Figure 3a**). The concentration of Cd in roots was higher than in shoots under Cd stress. However, Si addition significantly reduced Cd concentrations by 52.7% in roots and 21.5% in shoots of Cd-treated *P. glaucum* (**Figure 3b**).

**Figure 3:**
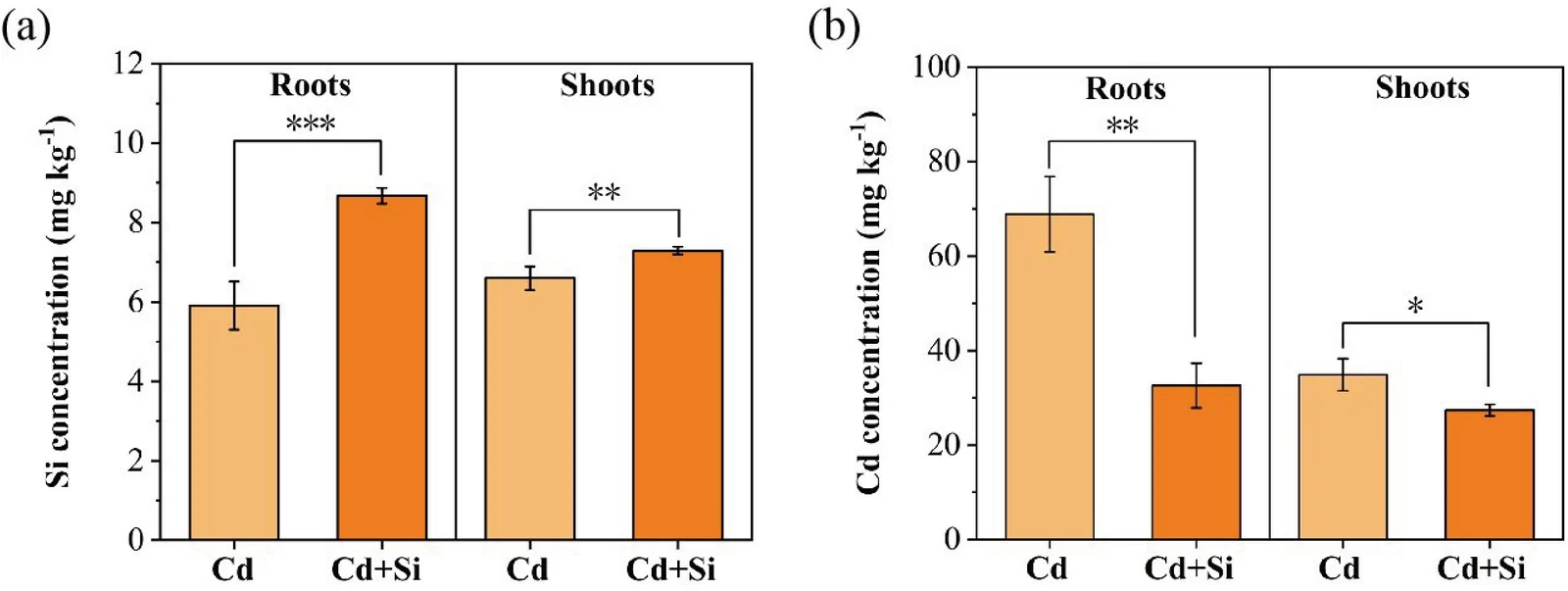
Effects of Si application on Si **(a)** and Cd concentration **(b)** in roots and shoots of *P. glaucum* under Cd stress. All values are presented as the mean ± standard deviation (n = 3), and differences between treatments are marked with asterisks (*p < 0.05, **p < 0.01, and ***p < 0.001).

### 3.5 Sequencing quality assessment and differentially expressed genes analysis

The average base percentages of the measured sample data reaching the Q30 and Q20 standards were both ≥96% (**Supplementary Table 3**). Principal component analysis (PCA) showed that Cd and Cd+Si treatments were separated from each other with significant differences (**Figure 4b**). In addition, a total of 6491 DEGs were identified from the sample group Cd-vs-Cd+Si (2062 genes were up-regulated and 4429 genes were down-regulated) (**Figure 4c**).

**Figure 4:**
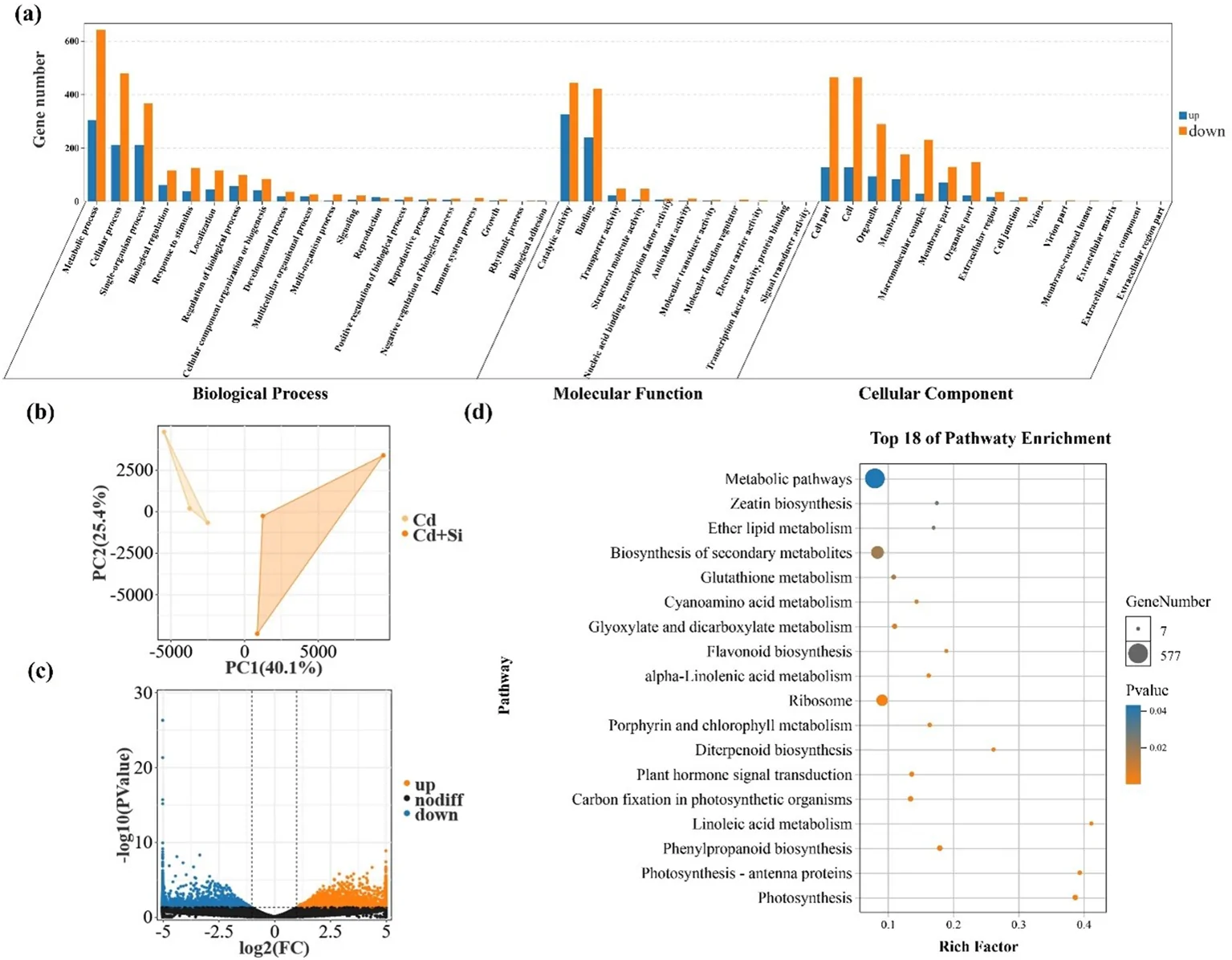
GO enrichment **(a)**, PCA **(b)**, volcano plot **(c)**, and KEGG pathway analysis **(d)** of DEGs. DEGS were selected by |log2-fold change| ≥ 1 and false discovery rate (FDR) < 0.05 **(c)**, with the Y-axis represents significant changes in gene expression, and the X-axis represents fold changes in gene expression. Blue and orange indicate up-regulated and down-regulated genes, respectively. Black indicates no differential genes. The Y- axis represents the KEGG pathway, and the X-axis represents the rich factor **(d)**. The rich factor means the enrichment degree of DEGs in each pathway. The dot size and color indicate the number and Pvalue of DEGs in the pathway, respectively.

### 3.6 GO enrichment and KEGG pathway analysis of DEGs

A total of 4710 annotated DEGs were divided into 43 level-2 functional categories, including 17 terms of biological process, 11 terms of molecular functions and 15 terms of cellular components (**Figure 4a**). In biological process, the majority of DEGs were involved in metabolic process, cellular process, and single-organism process. The terms of molecular functions were mainly related to catalytic activity and binding. In addition, most terms of cellular components were annotated in cell, cell part, and organelle, etc.

The biological function of DEGs was performed through KEGG pathway analysis, and a total of 18 pathways (*p* < 0.05) were significantly enriched (**Figure 4d**). The pathways related to photosynthesis include photosynthesis (ko00195), photosynthesis-antenna proteins (ko00196), and carbon fixation in photosynthetic organisms (ko00710) (**Supplementary Table 4**). In addition, 39 DEGs were enriched in the glutathione metabolic pathway (ko00480).

### 3.7 Genes related to photosynthesis, glutathione metabolism and heavy metal transport

Under Cd stress, the identified genes related to photosynthesis were all up-regulated (**Figure 5a**) after Si treatment. Among them, the expression of genes related to PS I including *PSAE* (Unigene0039749), *PSAF* (Unigene0040461), and *PsaD* (Unigene0071602) were increased 2.11-3.85 folds, and that of *PSBR* (Unigene0094379) and *PetC* (Unigene0074603) associated with PS II and Cyt b6/f was up-regulated 3.39-fold and 1.76-fold, respectively. In the glutathione pathway, multiple genes related to Glutathione S-transferases (GSTs) were differentially expressed (**Figure 5b**), and *GSTU10* (Unigene0038504), *GSTF1* (Unigene0051673), and *GSTU6* (Unigene0086352) were up-regulated (1.18-3.80 folds) in the Cd+Si treatment compared with the Cd treatment. However, genes related to heavy metal uptake and transport such as *ABCG36* (Unigene0062035), A*BCB11*(Unigene0008406), *Nramp5* (Unigene0064393), and *Nramp4* (Unigene0006465) were all significantly down-regulated (1.10-2.66 folds) in the Cd+Si treatment compared with the Cd treatment (**Figure 5c**).

**Figure 5:**
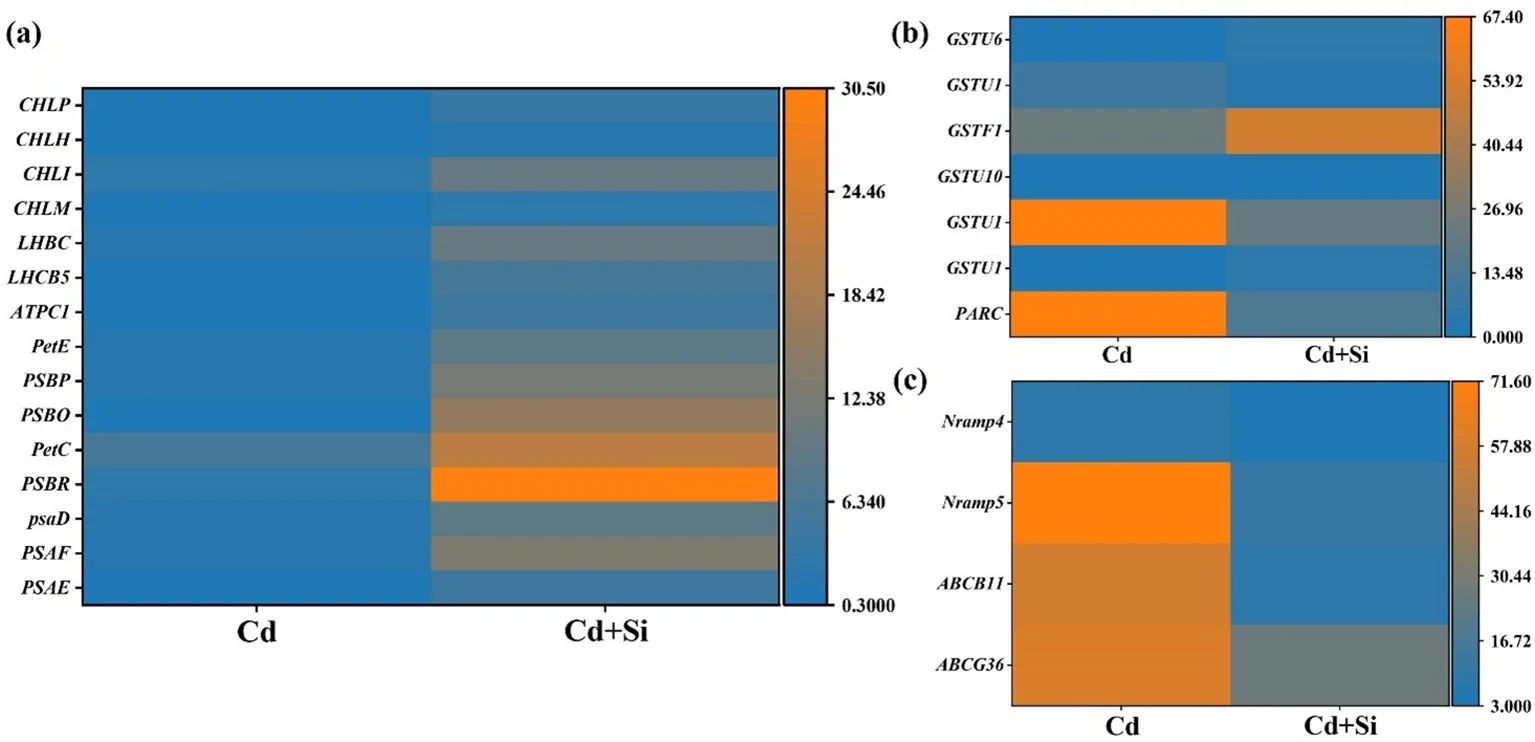
Effect of Si application on photosynthesis **(a)**, glutathione metabolism **(b)** and heavy metal uptake and transport-related genes expression **(c)** under Cd stress.

### 3.8 Transcriptome data validation

Eight DEGs (*ABCG36*, *Nramp5*, *Nramp4*, *ABCB11*, *MT3A*, *GSTU1*, *PSAF*, and *PSBR*) with GO functional annotation were randomly selected for qRT-PCR to verify the reliability of transcriptome data (**Supplementary Table 2**). The transcriptome data and qRT-PCR data showed similar expression trends (**Figure 6a**) and exhibited a significantly positive correlation (**Figure 6b**), indicating that the transcriptome data were reliable.

**Figure 6:**
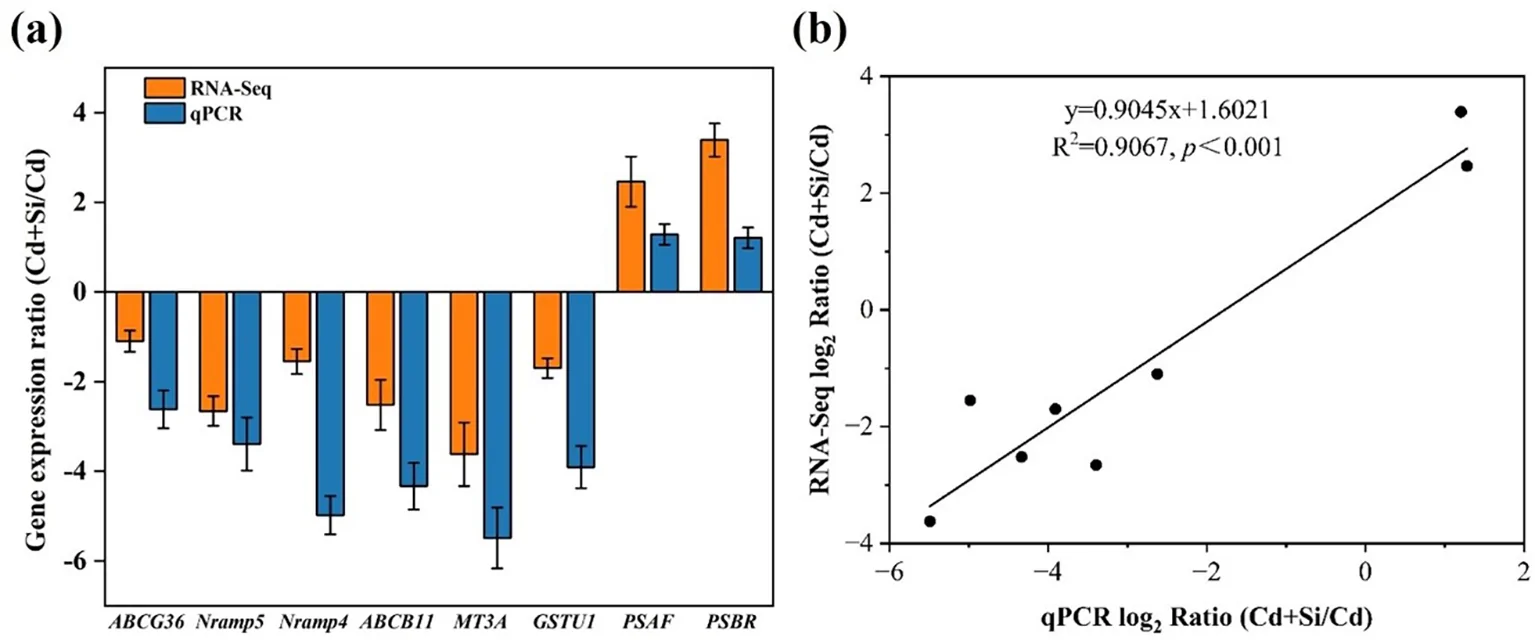
Consistency **(a)** and correlation analysis **(b)** of gene expression obtained by RNA-seq and qRT-PCR.

## 4 Discussion

The toxicity of the heavy metal Cd negatively affects physiological functions and inhibits plant growth. Our previous study found that Si could enhanced *P. glaucum* resistance against Cd stress (Pan et al., 2023). However, explaining the cause at the transcriptome level is insufficient. Therefore, it is crucial to explore the defense strategies and related molecular mechanisms of Si-induced plant resistance to Cd toxicity. In this study, we further employed root transcriptome sequencing to systematically dissect the molecular mechanisms underlying Si-mediated Cd tolerance in *P. glaucum* at the transcriptional level. By comparing gene expression between the Cd and Cd+Si treatments we identified differentially expressed genes involved in photosynthesis, glutathione metabolism (including *GST* family genes), and Cd uptake and transport-related genes. It is worth noting that based on our previous findings (Dong et al., 2019; Pan et al., 2023), this experiment only selected one Si concentration (2 mmol·kg^-1^) and one Cd stress level. The effectiveness of Si in alleviating Cd toxicity in plants may be influenced by multiple factors, including Si application concentration, Cd stress intensity, and plant growth stage. Therefore, future studies should incorporate multiple concentration gradients of both Si and Cd to more comprehensively evaluate the efficacy of Si in alleviating Cd toxicity in *P. glaucum*.

### 4.1 Si addition improves photosynthesis under Cd stress

Photosynthesis is the basic physiological process to ensure the survival of green plants. However, the adverse effects of Cd on plant photosynthesis are mainly manifested as interference with electron transfer during photosynthesis, inhibition of photosynthesis-related enzyme activities, and destruction of chloroplast structures (Perales-Vela et al., 2016; Ahmed et al., 2023). In our study, Si application increased the *P*_n_ of *P. glaucum* leaves under Cd stress (**Figure 1**). This result was similar to that reported by Jiang et al. (2023), who found that exogenous Si restored the photosynthetic performance of Cd-stressed wheat and induced reversibility of Cd toxicity. Furthermore, we observed that the application of Si contributed to the increase in chlorophyll content of *P. glaucum* under stress (**Table 1**), which promoted the improvement of light-use efficiency in plants. Previous studies have shown that the regulation of genes related to chlorophyll biosynthesis by Si addition can effectively reduce Cd toxicity in wheat (Wang et al., 2025). We also found that Si up-regulated the expression of Mg-chelatase CHL-related genes *CHLM* (Unigene0030702), *CHLP* (Unigene0059352), *CHLI* (Unigene0052433), and *CHLH* (Unigene0056173) under Cd stress, which promoted the synthesis of chlorophyll (**Figure 5**).

PSI and PSII are important parts of the plant electron transport chain (Mushtaq et al., 2024). In our study, Cd stress resulted in a significant reduction of *F*v/*F*_m_ and *F*v/*F*_0_ (**Table 1**), indicating an inhibition of light energy conversion of PSII (Drążkiewicz et al., 2003). However, Si induced an increase in *F*v/*F*_m_ and *F*v/*F*_0_ under Cd stress (**Table 1**), which contributed to maintaining the integrity of the photosynthetic system (Song et al., 2014). Gong et al. (2017) found that proteins related to PSI and PSII were significantly down-regulated under Cd stress. We found that Si significantly up-regulated the genes *LHCB5* (Unigene0040639) and *LHBC* (Unigene0050763) associated with LHCII protein under Cd stress (**Figure 5**). Song et al. (2014) also reported that Si could up-regulate genes *Os09g26810* and *Os04g38410* related to LHCII protein under zinc stress. In this study, Si application up-regulated the expression of *PetE* (Unigene0081293) related to plastocyanin under Cd stress, which contributed to electron transport in PSII and PSI (Zhang et al., 2018). These results indicated that Si enhanced the photosynthesis of *P. glaucum* under Cd stress by regulating photosynthesis-related genes.

### 4.2 Si enhances antioxidant defense system and glutathione metabolism under Cd stress

Excessive Cd leads to the burst of ROS and the imbalance of antioxidant enzyme system, thereby causing damage to plants (Yan et al., 2023). Activation of antioxidant enzymes in the ascorbic acid-glutathione (AsA-GSH) cycle can partially scavenge free radicals under stress (Riaz et al., 2022). Luo et al. (2024) reported that exogenous silicon application enhanced plant resistance to Cd-induced oxidative stress by increasing antioxidant enzyme activities and maintaining AsA-GSH homeostasis. In this study, we observed that Si application reduced the concentration of MDA and increased the activities of DHAR and MDHAR under Cd stress (**Figure 2**). This result was similar to the study by Chen et al. (2024), who found that foliar Si alleviated the oxidative damage of Cd to soybeans through increased antioxidant enzyme activities. Hasanuzzaman et al. (2017) also found that exogenous Si mediated Cd-induced oxidative stress in rape by modulating the AsA-GSH pathway.

The supergene enzyme family GSTs is encoded by multiple genes with different functions, and plays an important role in glutathione metabolism and maintenance of intracellular homeostasis (Altaf et al., 2022). Furthermore, GSTs catalyze the conjugation of GSH to heavy metals, and the formed complex is sequestered in the vacuole, contributing to cellular defense in Cd detoxification (Wang et al., 2021). Di et al. (2024) demonstrated that NH_4_^+^ activates the expression of antioxidant enzyme genes (*OsAPX2* and *OsCATA*) via the ABA-SAPK9-bZIP20-APX2/CATA transcriptional regulatory module, thereby enhancing Cd tolerance in rice. These findings underscore that transcription factor-mediated upregulation of antioxidant defenses constitutes an effective mechanism for combating Cd stress in plants. In our study, Si application up-regulated the genes *GSTU1* (Unigene0006510), *GSTU10* (Unigene0038504), *GSTF1* (Unigene0051673), and *GSTU6* (Unigene0086352) in Cd-stressed *P. glaucum* roots (**Figure 5**). Similarly, Zhou et al. (2016) found that exogenous Si application up-regulated the expression of the GST-encoding gene *GmGST21*, which contributed to the resistance of pakchoi to Cd stress. These results indicated that the expression trends of the *GST* family genes and other antioxidant-related genes identified in this study were highly consistent with those of homologous genes previously reported in rape and pakchoi, further suggesting that Si-induced enhancement of antioxidant enzyme activities and up-regulation of glutathione metabolism-related genes might be an important strategy to reduced Cd toxicity in *P. glaucum*. This consistency further suggests that Si enhances antioxidant enzyme activities and upregulates glutathione metabolism, which represents a key strategy for mitigating Cd toxicity in *P. glaucum*.

### 4.3 Si inhibits the expression of Cd uptake and transport-related genes under Cd stress

In Cd contaminated soil, Cd accumulates in plants through absorption and transport by the roots (Qi et al., 2020). Cai et al. (2022) found that Si application could reduce the concentration of Cd in shoots through the retention effect of Cd in rice roots. However, our results showed that Si application not only reduced the Cd concentration in shoots, but also reduced the Cd concentration in roots of *P. glaucum* under Cd stress (**Figure 3b**). Guo et al. (2022) found that water-soluble Si-Cd complexes in soil reduced the bioavailability of Cd, which was not conducive to the absorption and accumulation of Cd in rice. These results indicated that the inhibition of Cd uptake in roots of *P. glaucum* was an effective strategy to alleviate Cd stress.

Numerous studies reported that exogenous Si inhibited the uptake and transfer of Cd in plants through the regulation of specific genes (Luo et al., 2025). However, it is unknown whether Si affects the differential expression of genes related to heavy metal uptake and transport in *P. glaucum* roots from the transcriptome level. It is well known that Cd can enter plant cells through the ABC and NRAMP transporter family in roots (Shi et al., 2019; Zhou et al., 2022). In addition, the Nramp family is involved in the regulation and maintenance of metal ion homeostasis (such as Cd^2+^ and Al^3+^), and the ABC family is responsible for Cd efflux and vacuole sequestration in plants. Recently, Shen et al. (2021) showed that high expression of *Nramp5* resulted in an increased of Cd uptake in water spinach. Wu et al. (2016) found that knockout of the *Narmp5* gene significantly reduced Cd concentration in barley. In our study, Si significantly down-regulated the expression of *Nramp5* (Unigene0064393) in Cd-stressed *P. glaucum* roots (**Figure 5**). Similarly, Bao et al. (2021) revealed that downregulation of *OsNramp5* inhibited the efficient transport of Cd from rice roots to shoots. These results indicated that the expression trend of *Nramp5* identified in this study was consistent with that of previously reported Cd stress-responsive genes in other crops, and suggested that Si-mediated downregulation of *Nramp5* may be an important factor in reducing Cd uptake in *P. glaucum*. Furthermore, we also observed that Si inhibited the expression of *Nramp4* (Unigene0006465), *ABCG36* (Unigene0062035), and *ABCB11* (Unigene0008406), which might be beneficial for *P. glaucum* roots to reduce the absorption of Cd in soil (Fu et al., 2019). Therefore, exogenous Si may directly reduce Cd accumulation in *P. glaucum* by modulating the expression of genes involved in Cd uptake and root-to-shoot transport. However, because K_2_SiO_3_ is alkaline, its application can increase soil pH and shift exchangeable Cd to the residual fraction, thereby decreasing root Cd uptake (Pan et al., 2022). Therefore, future studies will incorporate pH-equilibrated treatments to isolate the specific role of Si.

## 5 Conclusion

This study deciphered the underlying mechanism of Si-mediated mitigation of Cd stress in *P. glaucum* through physiological and transcriptomic analysis (**Figure 7**). Si application improved plant photosynthesis-related traits and induced the up-regulation of photosynthesis related genes, which contributed to the growth of *P. glaucum* under Cd stress. Moreover, the addition of Si improved the activities of MDHAR and DHAR involved in the AsA-GSH cycle, and up-regulated the expression of key genes in the glutathione metabolic pathway, which alleviated oxidative stress in *P. glaucum* roots. And more importantly, Si significantly reduced Cd concentration in roots and shoots, and down-regulated the expression of Cd uptake and transport-related genes (such as *ABCG36*, *ABCB11*, *Nramp5*, and *Nramp4*), which may be an important reason for the reduction of Cd toxicity in *P. glaucum*. These results reveal silicon-mediated alleviation of Cd stress in *P. glaucum* and provide a potential strategy for reducing Cd accumulation in forage grasses grown on Cd-contaminated soils.

**Figure 7:**
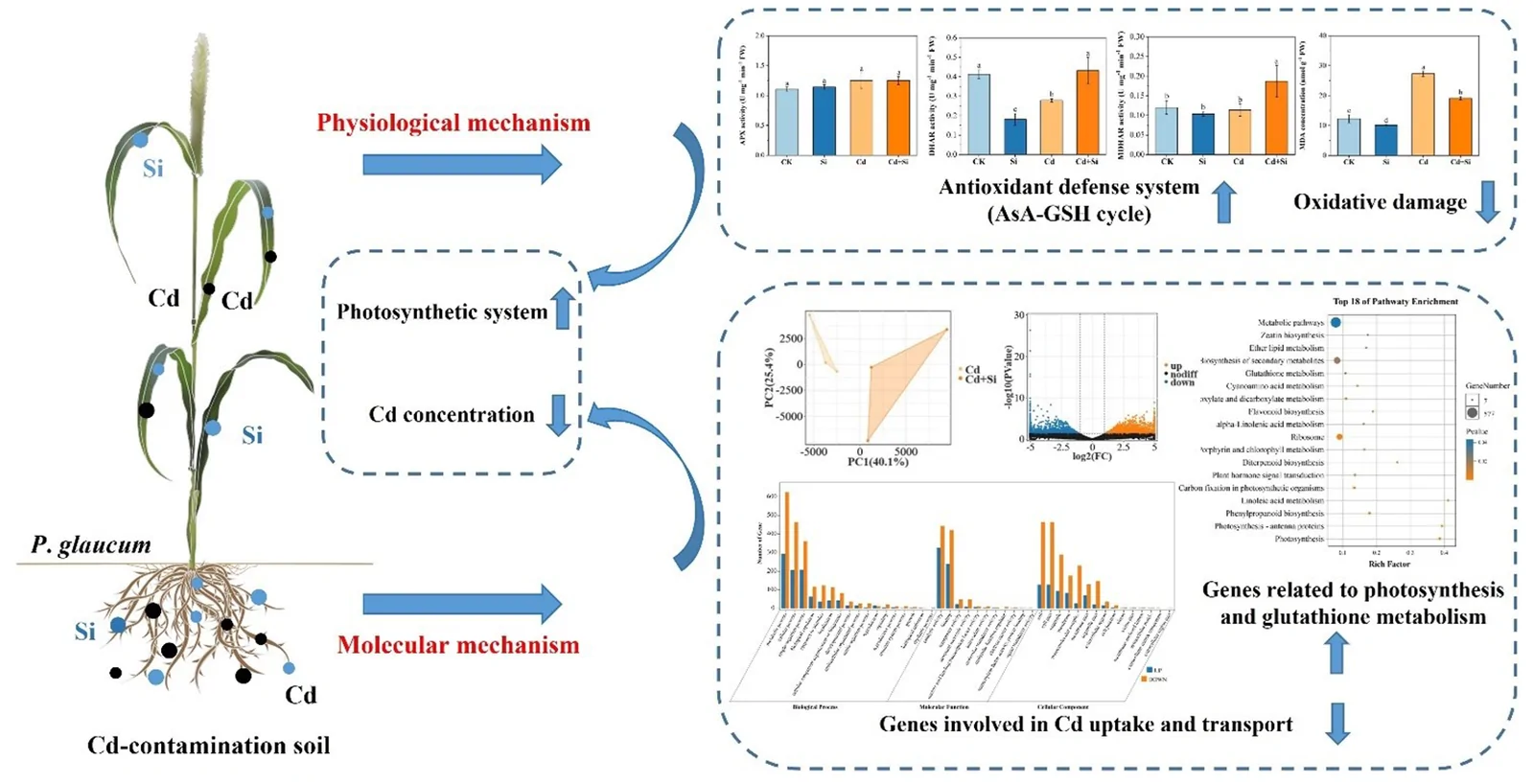
The underlying mechanism of silicon-mediated alleviation of cadmium stress in *P. glaucum* through physiological and transcriptomic analysis.

## Data Availability

The data presented in the study are deposited in the Mendeley Data. This data can be found here: https://data.mendeley.com/datasets/s3pxv23rkv/1.
