# Supplementary material for: Silicon-mediated alleviation of cadmium stress in Pennisetum glaucum: insights from physiology and root transcriptomics analysis
Source: Front Plant Sci. 2026 Oct 13;17:1916768. doi: 10.3389/fpls.2026.1916768 (PMC13710062; doi:10.3389/fpls.2026.1916768)
Supplement: Supplementary file 1 [file Table1.docx]

Supplementary Material

## 1.1 Supplementary Tables

**Supplementary Table S1** The basic soil chemical properties.

| pH | Organic matter  (g kg^-1^) | Total N  (g kg^-1^) | Total P  (g kg^-1^) | Total K  (g kg^-1^) | Available N  (mg kg^-1^) | Available P  (mg kg^-1^) | Available K  (mg kg^-1^) |
| --- | --- | --- | --- | --- | --- | --- | --- |
| 5.58 | 111.32 | 1.50 | 0.21 | 11.66 | 155.34 | 57.30 | 174.10 |

**Supplementary Table S2** List of primers used for qRT-PCR experiments.

| Symbol | Gene ID |  | 5'-3' |  |
| --- | --- | --- | --- | --- |
| *ABCG36* | Unigene0062035 | F | CGGTGGACAAAGAAAGCGTG | Program: 90 s for 95 °C, followed by 40 cycles of 5 s for 95 °C, 15 s for 60 °C, 20 s for 72 °C, and finally 5 min for 72 °C. |
|  |  | R | CCGAGGATGTGGATGGACTG |  |
| *Nramp5* | Unigene0064393 | F | TCCAAGTTTGTTTAGCCTTTCACC |  |
|  |  | R | GCCAGGATGAAGGTAGGGTA |  |
| *Nramp4* | Unigene0006465 | F | TCCGACAGGGCAAGTCATAG |  |
|  |  | R | GCAGCAATCACACCAACCTC |  |
| *ABCB11* | Unigene0008406 | F | TCCAAGCCGTCCAGATGTTC |  |
|  |  | R | CTGCCACTCTCTCCCACAAG |  |
| *MT3A* | Unigene0044474 | F | GGAGCCAGTGCACGAAGA |  |
|  |  | R | GACAACCTCCTCCTCACGG |  |
| *GSTU1* | Unigene0023563 | F | ACTTCTGGGTGAGCCCGTTC |  |
|  |  | R | TTGGAGCGGAGGAGGAGGT |  |
| *PSAF* | Unigene0040461 | F | GCCTGCTCTTCCTCTACATC |  |
|  |  | R | GACGAGGTCTCCGTTGATGA |  |
| *PSBR* | Unigene0094379 | F | ATCAAGACTGACAAGCCCTACG |  |
|  |  | R | GGTTTTCCCGCCGACATAGA |  |
| *Action* |  | F | CGTGACCTTACCGACAACCT |  |
|  |  | R | CCACCACTGAGGACGACATTAC |  |

Note: *ABCG36*, ABC transporter G family member 36; *Nramp5*, Metal transporter Nramp5; *Nramp4*, Metal transporter Nramp4; *ABCB11*, ABC transporter B family member 4; *MT3A*, metallothionein-like protein 3A; *GSTU1*, *probable glutathione S-transferase GSTU1*; *PSAF*, Photosystem I reaction center subunit II; *PSBR*, photosystem II 10 kDa polypeptide; F: Forward primer; R: Reverse primer.

**Supplementary Table S3** Quality control of datas.

| Samples | Raw reads | Clean reads | Q30 (%) | Q20 (%) |
| --- | --- | --- | --- | --- |
| Cd-1 | 54270838 | 53048430 | 96.09 | 98.77 |
| Cd-2 | 51346058 | 50158152 | 96.18 | 98.79 |
| Cd-3 | 58871512 | 57573238 | 96.07 | 98.76 |
| Cd+Si-1 | 57978504 | 56606168 | 96.00 | 98.74 |
| Cd+Si-2 | 62405112 | 60918708 | 96.04 | 98.75 |
| Cd+Si-3 | 58236942 | 56860444 | 96.08 | 98.77 |

**Supplementary Table S4** Effect of Si on photosynthesis and glutathione metabolism-related pathways under Cd stress.

|  | Pathway | Candidate genes with pathway annotation (Ratio) | P value | Pathway ID |
| --- | --- | --- | --- | --- |
| 1 | Photosynthesis | 60 (4.38%) | 0.000000 | ko00195 |
| 2 | Photosynthesis - antenna proteins | 26 (1.90%) | 0.000000 | ko00196 |
| 3 | Carbon fixation in photosynthetic organisms | 55 (4.02%) | 0.000023 | ko00710 |
| 4 | Glutathione metabolism | 39 (2.85%) | 0.014595 | ko00480 |
